# Case of Poisoning by Chloroform Taken Internally

**Published:** 1857-04

**Authors:** James Spence


### ARTICLE XIII.

*Case of Poisoning by Chloroform taken internally.* By James Spence, Esq.

On the 19th of May last, at a quarter past 10, P. M., I was called to see A. B——, aged twenty-one, one of the female servants of this hospital, who, I was informed, had twenty minutes previously swallowed two ounces of pure chloroform. I found her lying in bed, half dressed, in a state of perfect unconsciousness, (apparently in a profound sleep,) from which she could not be roused. Her breath did not smell of chloroform. Pupils very much contracted; conjunctiva quite insensible; body of normal temperature; respiration tranquil and regular; pulse 78, soft and tolerably full; no congestion of face. I immediately ordered sinapisms to be applied to the extremities and over the epigastrium, and having secured the able assistance of my colleague, Dr. Thorburn, proceeded to evacuate the stomach by the stomach-pump, it being impossible to make her swallow an emetic. A delay of nearly ten minutes occurred before the stomach-pump was procured. When it was applied, the matters evacuated had not the slightest odor of chloroform, nor of opium, which was suspected from the excessively contracted state of the pupils. About half an ounce of mustard was introduced into the stomach, which was again emptied, and then a drachm of aromatic spirit of ammonia, with one ounce of brandy, administered by means of the stomach-pump. Some feeble attempts at vomiting ensued, and the pupils became fully dilated, and continued so for some minutes, but still continued quite immovable when exposed to a strong light. At the same time the beats of the pulse and number of respirations slightly increased in frequency, but shortly after fell below their previous standard. A powerfully stimulating enema was now administered, and, after the lapse of ten minutes, respiration becoming slow and stertorous, the pulse at the same time sensibly flagging, and the face becoming livid and congested, galvanism was resorted to, a free circulation of air being kept up around the patient, and her tongue held forward by a pair of catch forceps to prevent closure of the glottis. The number of respirations, however, continued to decrease, falling so low as seven in the minute, and, accordingly, an additional pair of plates were added to the galvanic battery, greatly increasing its strength and efficiency, while enemata of beef-tea and brandy were administered frequently. Dr. William Gairdner, one of the visiting physicians to the hospital, had been sent for, and arrived about twenty minutes past eleven, P. M. He recommended the administration of a large black draught, which was done by means of the stomach-pump. This produced severe retching and attempts to vomit, during which the patient was repeatedly almost asphyxiated. Keeping up artificial respiration with the aid of galvanism was now evidently our only resource, and this was continued, with occasional short intermissions, for nearly two hours. Stimulating enemata were given every half hour, and warmth applied to the extremities, which became excessively cold. Everything, however, appeared of no avail, and respiration fell to two per minute; the pulse at the wrist became imperceptible, while the face and neck were perfectly livid. At one time, indeed, breathing ceased altogether for nearly two minutes, and the jaw fell. The remedial measures were, however, persevered in, and in about half an hour we had the gratification of perceiving some sign of amendment. Her pulse gradually gained in strength, while her breathing became less embarrassed, *her breath now smelling strongly of chloroform*—Half-past two, P. M. Pupils became widely dilated, the sensibility of the conjunctiva returning, and the lividity of the face disappeared. Galvanism was now desisted from, although the patient still remained unconscious, all attempts to rouse her being unavailing. Three A. M. Bowels very freely purged; pulse 94, gaining strength; respiration 28 per minute; the extremities have recovered their natural temperature. Half-past three A. M. Consciousness slowly returning. Four A. M. For the first time the patient answered when addressed, and of her own accord opened her eyes. The white of egg beat up with mucilage and warm milk was now cautiously administered, and attendants were directed to watch her carefully.

May 20th—Ten, A. M.—Perfectly sensible; pulse 100, soft; respiration unembarrassed, and not hurried in any marked degree; complains of general pain in abdomen, of thirst, and great nausea; tongue moist, but is considerably swollen and very painful. Hot fomentations applied over abdomen, and she was ordered to have five minims of tincture of opium, every three hours, in half an ounce of mucilage. Has not passed any urine since last night; bladder empty. Evening. Tongue moist, and still extremely painful; pulse 120, soft and regular; general pain over abdomen; has been severely purged, and a considerable quantity of blood passed by stool; urine passed freely; complains of a dull aching pain across the loins. To continue the fomentations, have a starch enema containing half a drachm of tincture of opium, and to swallow pieces of ice occasionally.

21st.—No return of the diarrhea; slept a little during the night; pulse 132, soft; tongue furred; thirst excessive; pain is now entirely referred to the epigastrium, and is increased by pressure, which also induced a tendency to vomit; feels drowsy, and pupils are slightly contracted; urine passed abundantly. To apply twelve leeches to the epigastrium and a sinapism along the spine. Evening—Much relieved; pulse 130; tongue moist; less drowsy, and free from nausea; diarrhea has recurred, but not severely. To repeat the starch and opium enema.

22d.—Greatly better; pulse 100; complains merely of a general feeling of soreness; has taken a little beef-tea, which was retained in the stomach.

23d.—Doing well; pulse 90, soft.

25th.—Is able to sit up, and the following day returned to her work.

I have communicated the particulars of this case from its great interest, being, as far as I am aware, the only one on record of poisoning by chloroform administered internally. The only other case I know of its occurrence happened also in this hospital, some years ago, when a patient, having surreptitiously got possession of a bottle of choloroform, swallowed (if I remember rightly) the enormous quantity of six ounces. The man recovered from the immediate effects of the poison under the use of stimuli and galvanism, but died in great agony, within forty-eight hours, with symptoms of acute gastritis. When first called to the present case, I should certainly have thought it a case of poisoning from opium had I not been shown the bottle which had contained the chloroform, the contracted state of the pupils, coupled with the patient's complete insensibility, strongly resembling the effects produced by the former drug. The diminution of the frequency of respiration, however, was not proportionate to the amount of stupor. The indications for treatment were evidently to sustain the flagging vital power by stimulants and galvanism: but I am doubtful of the propriety in such cases of administering alcoholic stimuli, which might tend to aggravate the symptoms; and, should I ever meet with a similar case, I should trust more to the preparations of ammonia, as we are, I think, justified in supposing that chloroform, to a certain extent at least, acts by causing an excess of carbon in the blood, which would be still further increased by the administration of any form of alcohol. In fact, the patient's condition was precisely that of extreme drunkenness. It is worthy of notice that, although certainly not more than forty minutes elapsed from the time the chloroform was swallowed till the stomach was evacuated by the stomach-pump, no smell of chloroform was appreciable in the contents of the stomach. This could have arisen only from extremely rapid absorption of the poison, or from its having quickly passed into the small intestines, and been thence absorbed more gradually. The latter supposition is favored by the fact that a strong odor of chloroform was perceived in the patient's breath when she began to rally from its effects, nearly four hours subsequently to its administration, although it could not be detected before. It was from a consideration of this kind that Dr. Gairdner prescribed an active cathartic, in hopes of emptying the intestines of their noxious contents. It is still a disputed point whether the action of chloroform on the nervous centres affects primarily the respiratory or circulatory systems. The former is maintained by Mr. Bickersteth, of Liverpool, who has supported his arguments by several interesting and carefully conducted experiments, while, in the case of death from inhalation of chloroform recorded by Dr. Dunsmure, the heart appeared to cease to beat before the respiratory movements were suspended; and a similar observation was made in the case lately published by Dr. Mackenzie, of Kelso. In the case before us, the heart and lungs seemed to flag *pari passu*—certainly the radial pulse disappeared before respiration was entirely arrested, but unfortunately at the moment it was not observed if the heart had likewise stopped.

The successful result of this case may serve to encourage medical men to persevere, even against hope, under similar circumstances, in continuing their exertions. Mr. Lowe, two or three years ago, published a case of inhalation in which respiration and the heart's action were arrested for fully four minutes when under continued artificial respiration; the *pulse first* slowly reappeared, followed by a return of the natural respiratory movements.

In cases, however, where chloroform has been swallowed, it is not only the immediate effects of the drug that we have to fear, and this is well exemplified in the instance of the patient already quoted, who died from the subsequent inflammation set up. Fortunately, in the present case, the symptoms of the secondary danger were never very severe, and were easily controlled by mild remedies.—*Lancet*, Aug. 9th, 1856.

